# Orbital Diffuse Large B‐Cell Lymphoma Masquerading as Periorbital Cellulitis in a Paediatric Patient With Ataxia‐Telangiectasia: A Case Report

**DOI:** 10.1155/crom/7092035

**Published:** 2026-11-09

**Authors:** Fajer Alabdulrazzaq, Arwa Alkanderi, Bashayer AlHazeem, Abdullah Alenezi

**Affiliations:** ^1^ Department of Paediatrics Farwaniya Hospital Ministry of Health (MOH) Farwaniya Kuwait moh.gov.rw; ^2^ Kuwait Institute for Medical Specializations (KIMS) Kuwait Ministry of Health (MOH) Kuwait City Kuwait

**Keywords:** ataxia-telangiectasia, dacryocystitis, diagnostic delay, non-Hodgkin lymphoma, ocular adnexal lymphoma, primary immunodeficiency

## Abstract

Ataxia‐telangiectasia (A‐T) is a rare autosomal recessive disorder characterised by cerebellar ataxia, combined immunodeficiency and elevated predisposition to lymphoid malignancy, rendering orbital malignancy clinically and radiologically indistinguishable from infection. We report a 7‐year‐old female with A‐T presenting with left periorbital cellulitis unresponsive to prolonged antimicrobial therapy, surgical drainage and antifungal escalation. Incisional biopsy of a persistent periorbital mass revealed diffuse large B‐cell lymphoma (DLBCL), confirmed on immunohistochemistry. Staging indicated Lugano‐modified Ann Arbour Stage IV disease, with a primary orbital mass and non‐contiguous mediastinal and abdominal disease on cross‐sectional imaging. Bone marrow assessment showed no morphological involvement, although flow cytometry detected a light chain–restricted B‐cell population. The pulmonary lesions could not be radiologically distinguished from necrotising infection. She received R‐CHOP (rituximab, cyclophosphamide, doxorubicin, vincristine and prednisolone) with cyclophosphamide and vincristine reduced to approximately 50% of standard doses but died approximately 2 months after diagnosis, following a severe necrotising pulmonary infection after the second treatment cycle. This case underscores the importance of early malignancy consideration in immunocompromised A‐T patients with refractory orbital disease, the critical role of timely tissue biopsy and the diagnostic difficulty of distinguishing lymphomatous involvement from infection on imaging alone in this population.

## 1. Introduction

Ataxia‐telangiectasia (A‐T) is a rare autosomal recessive multisystem disorder characterised by progressive cerebellar ataxia, oculocutaneous telangiectasias, combined immunodeficiency and marked radiosensitivity. The multisystem manifestations of A‐T stem from biallelic loss‐of‐function mutations in the ATM gene, impairing DNA repair, cell cycle checkpoint control and apoptosis, leading to progressive genomic instability [1, 2]. The resulting immunodeficiency renders affected children susceptible to recurrent sinopulmonary infections that may extend to the orbit, producing periorbital cellulitis and abscess clinically and radiologically indistinguishable from malignant infiltration [2–6].

Malignancy is among the most feared complications of A‐T, with non‐Hodgkin lymphoma (NHL) accounting for up to 55% of A‐T‐associated cancers, characteristically manifesting as high‐grade B‐cell disease at a median age of 9.7 years, with advanced‐stage presentation and extranodal involvement in up to 75% of cases [2, 7]. Orbital NHL in paediatric A‐T is exceedingly rare, with only isolated reports in the literature [8]. Diagnosis is compounded by relative contraindication to ionising radiation limiting imaging to magnetic resonance imaging (MRI), and close clinical mimicry of orbital infection, frequently delaying histopathological confirmation [4, 6, 9].

We present a 7‐year‐old female with A‐T diagnosed with orbital diffuse large B‐cell lymphoma (DLBCL) following prolonged treatment‐refractory periorbital infection, to highlight the diagnostic pitfalls of this presentation and the critical role of biopsy when orbital infection fails to respond to antimicrobial therapy.

## 2. Case Presentation

### 2.1. Patient Background

A 7‐year‐old female with established A‐T presented with a neurological baseline of cerebellar ataxia, generalised hypotonia, gaze‐evoked nystagmus and gait impairment requiring support. She received monthly intravenous immunoglobulin (IVIG) at 0.8 g/kg and prophylactic trimethoprim‐sulfamethoxazole (TMP‐SMX) 3 mg/kg/dose three times weekly, with her most recent IVIG dose 6 weeks prior to admission. Family history was unremarkable.

### 2.2. Presenting Illness

In spring 2026, she presented with a 2‐week history of left medial canthal swelling with mild erythema, epiphora, high‐grade fever and productive cough. Oral amoxicillin‐clavulanate (45 mg/kg/day), prescribed at a private clinic, failed to achieve improvement; she was subsequently escalated to ambulatory intravenous ceftriaxone for 7 days, during which TMP‐SMX prophylaxis was withheld. Fever recurred within 2 days of completing the course, and periorbital swelling persisted, prompting emergency department presentation and admission.

On admission, she was febrile at 39.6°C with marked pallor. Chest auscultation revealed bilateral coarse crepitations. Cardiovascular, abdominal and neurological examinations were unchanged from baseline. Oculofacial examination demonstrated left medial canthal swelling with epiphora and tenderness over the lacrimal sac; the right eye was normal. Formal ophthalmological assessment confirmed orthophoria, full extraocular motility without diplopia, a clear cornea, white conjunctiva, deep anterior chamber and reactive pupils bilaterally, with left lower lid blepharitis. Otoscopic examination revealed bilaterally dull tympanic membranes, confirmed on tympanometry as bilateral Type B curves consistent with serous otitis media. Nasal passages were clear. She was admitted with a clinical impression of left acute dacryocystitis with ipsilateral preseptal cellulitis and concurrent systemic infection/pneumonia (Figure 1).

**Figure 1:**
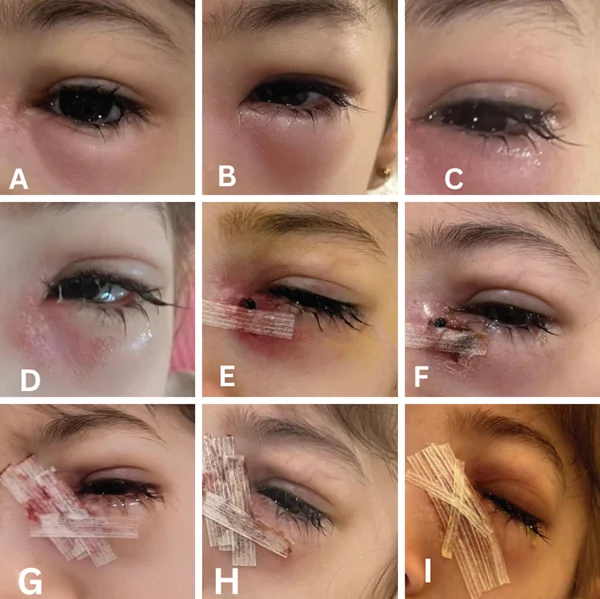
Sequential periorbital photographs illustrating the clinical evolution of left medial canthal swelling from admission (Day 0) to diagnosis. (A) Day 2: persistent left medial canthal erythema and swelling despite initiation of intravenous piperacillin–tazobactam; clinical impression of acute dacryocystitis with preseptal cellulitis. (B) Day 3: ongoing periorbital oedema and erythema with no clinical improvement. (C) Day 5: transient apparent reduction in swelling and erythema. (D) Day 8: marked periorbital swelling with worsening erythema; MRI orbit performed confirming medial extraconal phlegmon; multidisciplinary team convened. (E) Day 9: postoperative Day 0 following incision and drainage of lacrimal abscess and FESS; wound dressing in situ. (F) Day 11: postoperative Day 2; persistent periorbital swelling with wound dressing in situ, fever recurred. (G) Day 15: postbiopsy Day 1, following repeat incision and biopsy on hospital Day 14. (H) Day 16: persistent periorbital swelling with wound dressing in situ; histopathological result pending. (I) Day 17: primary histopathology of the biopsy specimen confirmed B‐cell non‐Hodgkin lymphoma, with DLBCL confirmed on subsequent reference review; oncology referral initiated. MRI = magnetic resonance imaging; FESS = functional endoscopic sinus surgery; DLBCL = diffuse large B‐cell lymphoma.

### 2.3. Initial Investigations and Management

The patient was placed in a protective isolation room. Initial laboratory evaluation demonstrated mild leukocytosis (15.2 × 10^3^/*μ*L), anaemia (haemoglobin 8.8 g/dL) with concurrent hypoferremia (serum iron 3 *μ*mol/L), and markedly elevated inflammatory markers (C‐reactive protein [CRP] 103 mg/L; erythrocyte sedimentation rate [ESR] 85 mm/h). Blood and urine cultures yielded no growth. Empiric intravenous piperacillin–tazobactam (300 mg/kg/day every 8 h) was initiated for systemic coverage, alongside topical moxifloxacin drops four times daily and dexamethasone/neomycin/polymyxin B ointment at night. IVIG (0.8 g/kg) was administered on hospital Day 4 to address the delayed maintenance dose.

### 2.4. Imaging and Surgical Intervention

Despite continued empiric therapy and persistently negative cultures, the patient failed to improve clinically. MRI of the brain and orbit (Figure 2) performed on hospital Day 8 demonstrated complicated sinusitis with left periorbital cellulitis and a medial extraconal phlegmon measuring 2.4 × 2.1 × 2.5 cm. This prompted the convening of a multidisciplinary team including paediatric immunology, ophthalmology and otolaryngology, and the patient subsequently underwent emergent combined surgical intervention on hospital Day 9: incision and drainage of the lacrimal abscess by oculoplastics, and concurrent functional endoscopic sinus surgery (FESS) by the ear, nose and throat (ENT) team for source control of the underlying sinusitis.

**Figure 2:**
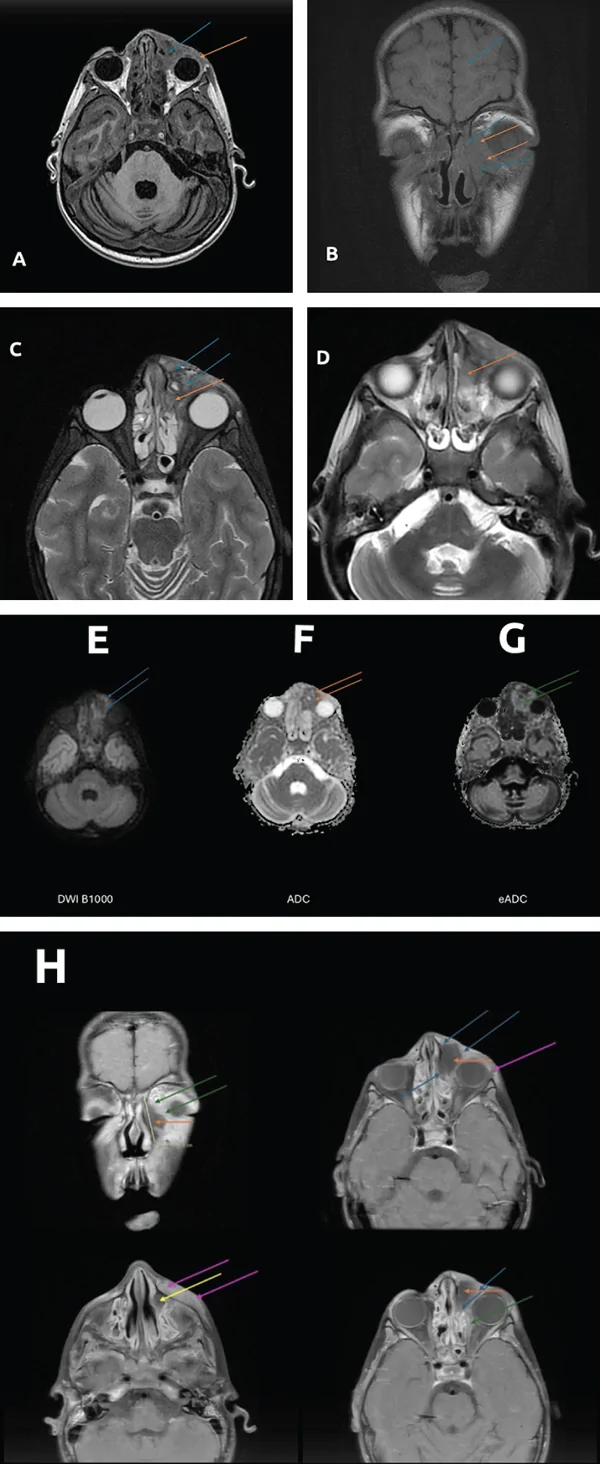
Magnetic resonance imaging of the orbits. (A) Axial T1‐weighted image demonstrates an ill‐defined area of abnormal low T1 signal intensity centred at the medial canthus/lacrimal sac of the left orbit (blue arrow), with associated preseptal oedema (orange arrow). (B) Coronal T1‐weighted image shows erosion of the ipsilateral lamina papyracea/medial orbital wall (blue arrow) and extraconal involvement with effacement of the surrounding fat planes (orange arrow). (C,D) Axial T2‐weighted images redemonstrate the abnormal intermediate‐to‐low T2 signal intensity at the medial canthus/lacrimal sac of the left orbit (blue arrow), with involvement of the left anterior ethmoid air cells (orange arrow). (E) DWI B1000, (F) ADC, and (G) eADC images demonstrate diffusion restriction within the phlegmonous collection: high signal intensity on DWI B1000 (blue arrow, [E]) and eADC (green arrow, [G]) with corresponding low signal intensity on ADC (orange arrow, [F]). (H) T1 fat‐saturated postcontrast images reveal a thick‐walled, peripherally enhancing collection with a central nonenhancing component (orange arrow), involvement of the left extraconal fat planes (green arrows), encasement of the proximal nasolacrimal duct (yellow arrow) and subcutaneous preseptal oedema (pink arrow). T1 = T1‐weighted; T2 = T2‐weighted; DWI = diffusion‐weighted imaging; ADC = apparent diffusion coefficient; eADC = exponential apparent diffusion coefficient.

### 2.5. Antimicrobial Escalation

Following a brief postoperative improvement, the patient developed recurrent febrile spikes on hospital Day 11. Repeat soft‐tissue ultrasound on the same day revealed an ill‐defined heteroechoic subcutaneous collection measuring 1.9 × 1.4 cm at the left medial canthus extending to the lower eyelid, consistent with abscess recollection. In the setting of persistently negative bacterial and fungal cultures, antimicrobial coverage was broadened to address potential opportunistic pathogens. Empirical antifungal therapy was initiated with caspofungin (70 mg/m^2^) on hospital Day 11, then escalated to liposomal amphotericin B (5 mg/kg/day) on hospital Day 14 to broaden antifungal coverage. Concurrently, piperacillin–tazobactam was switched to 6‐h dosing (300 mg/kg/day), and TMP‐SMX was commenced at therapeutic dosing (8 mg/kg/day).

### 2.6. Biopsy and Diagnosis

With persistent periorbital swelling and ongoing febrile episodes, repeat incision and biopsy was planned on hospital Day 12 and performed on hospital Day 14. Postbiopsy MRI demonstrated a residual left medial canthus extraconal collection with interval enlargement to 4.2 × 2.1 × 2.9 cm and diffusion restriction, consistent with disease progression.

Histopathological examination of the incisional biopsy specimens (labelled A and B, inferior to the medial canthal tendon and medial canthal ligament, left eye) revealed multiple tissue fragments composed of sheets of atypical lymphoid cells infiltrating the ocular muscles, with marked tumour necrosis. The cells were medium‐to‐large, with scant‐to‐moderate amphophilic to clear cytoplasm, moderate nuclear atypia, round‐to‐oval vesicular nuclei, occasional prominent nucleoli, frequent mitoses and frequent apoptotic figures. Initial immunohistochemistry at the primary laboratory showed tumour cells positive for CD20, CD79a (weak), BCL‐2, CD99 and CD43, and negative for CD3, CD10, CD23, CD34, TdT, MPO, pan‐cytokeratin (AE1/AE3), desmin and synaptophysin; BCL‐6 staining was noncontributory, and the Ki‐67 proliferative index was approximately 80%. These findings were compatible with non‐Hodgkin B‐cell lymphoma, and the case was referred to the national cancer centre for confirmatory ancillary studies and definitive subtyping. Confirmatory review with an expanded immunohistochemical panel demonstrated diffuse positivity for CD20, CD79a, BCL‐2, MUM‐1 and PAX‐5, with focal/weak positivity for CD99 and CD43; tumour cells were negative for CD3, CD5, CD10, BCL‐6, CD21, CD23, TdT, CD30, CD15, ALK, EMA, MPO, CD1a, CD34, pan‐cytokeratin, synaptophysin, desmin and HHV‐8, with CD3/CD43/CD5 highlighting admixed reactive T‐lymphocytes and CD68 highlighting scattered histiocytes. The Ki‐67 proliferative index was approximately 80%. MYC, BCL2 and BCL6 rearrangement studies, MYC immunohistochemistry and Epstein–Barr virus (EBV) studies (immunohistochemistry and EBV‐encoded RNA [EBER] in situ hybridisation) could not be obtained (see Limitations). This immunophenotypic and morphological profile was diagnostic of DLBCL, classified on reference review as an inborn error of immunity–associated lymphoma.

### 2.7. Staging and Oncological Referral

Following family counselling, antimicrobials were discontinued and the patient was transferred to a specialised paediatric haematology‐oncology centre for staging and definitive management, on maintenance TMP‐SMX. Staging investigations revealed the following: chest radiograph showed bilateral lower and midzone opacification with mediastinal widening (Figure 3A). Contrast‐enhanced computed tomography (CT) of the neck, chest, abdomen and pelvis demonstrated multiple bilateral, variable‐sized, predominantly centrally necrotic pulmonary masses (index lesions measuring up to 3.9 × 3.4 cm in the left lower lobe, with internal cavitation and 3.4 × 3.7 cm in the right lower lobe; Figure 3B,C), enlarged mediastinal lymph nodes, prominent but subcentimetre axillary nodes (up to 5 mm in short axis), soft tissue density at the porta hepatis and along the left splenic vein and a small enhancing nodule at the umbilicus (1.3 × 0.6 cm); the kidneys were bulky bilaterally without discrete lesions, and a mild amount of pelvic free fluid was noted. The radiologist explicitly noted that, although the appearances could suggest lymphomatous pulmonary involvement given the known history, widespread necrotising pulmonary infection could produce a similar radiological picture in this immunocompromised patient, and recommended clinical and laboratory correlation.

**Figure 3:**
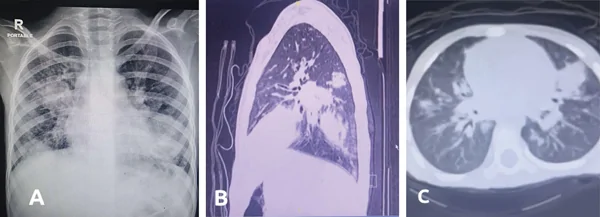
Staging imaging of the chest. (A) Portable anteroposterior chest radiograph showing bilateral lower and midzone opacification with mediastinal widening. (B) Coronal CT of the chest in lung window demonstrating extensive bilateral pulmonary consolidation and masses with areas of cavitation, and enlarged mediastinal lymph nodes. (C) Axial CT of the chest in the lung window showing bilateral pulmonary masses with surrounding ground‐glass opacification; lymphomatous involvement could not be radiologically distinguished from necrotising infection. CT = computed tomography.

Bone marrow aspiration was cellular and particulate, with mild hypocellularity for age and a myeloid‐to‐erythroid ratio of 2.8:1. Morphological examination demonstrated dysplastic erythropoiesis, including megaloblastoid changes, irregular nuclear contours, nuclear fragmentation and multinucleated forms; granulopoiesis was left‐shifted, and blasts were not increased (1%). Occasional small atypical lymphocytes of uncertain significance were noted, but no morphologically overt lymphoma cells were identified. Trephine biopsy confirmed hypocellular marrow for age (overall cellularity approximately 60%) with preserved trilineage haematopoiesis, moderately increased megakaryocytes and scattered macrophages with haemophagocytosis; no definite marrow infiltration was seen on histology. However, flow cytometric immunophenotyping of the aspirate identified a lambda light chain–restricted B‐cell population (CD19+ cells 15% of the lymphocyte gate; lambda 85% vs. kappa 13%; CD10 and CD5 negative), reported as suspicious for a B‐cell lymphoproliferative disorder. Marrow involvement was therefore demonstrated by flow cytometry only and was not established morphologically. Bone marrow cytogenetic analysis showed a normal female karyotype (46,XX) with structural chromosomal aberrations (breaks) in 20% of metaphases analysed, consistent with the underlying genomic instability of A‐T.

Cerebrospinal fluid (CSF) cytology demonstrated predominantly degenerated cells with a few well‐preserved lymphocytes and monocytes; no viable atypical cells were identified, though the nature of the degenerated cell population could not be further characterised. Positron emission tomography (PET) was deferred due to financial constraints. At transfer to the haematology‐oncology centre, CRP remained markedly elevated (205.6 mg/L), and pretreatment lactate dehydrogenase (LDH) was elevated (335 U/L). The disease was staged as Lugano‐modified Ann Arbour Stage IV, on the basis of a primary extranodal orbital mass with non‐contiguous nodal and soft tissue disease on both sides of the diaphragm (mediastinal lymphadenopathy; soft tissue disease at the porta hepatis and along the splenic vein).

### 2.8. Management and Outcome

The patient received care at a specialised paediatric haematology‐oncology centre. Chemoimmunotherapy with R‐CHOP (rituximab, cyclophosphamide, doxorubicin, vincristine and prednisolone) was administered in 3‐weekly cycles with interim response review. Cyclophosphamide and vincristine were dose‐reduced to approximately 50% of standard dosing, whereas rituximab, doxorubicin and prednisolone were administered at standard doses. Before the first cycle, baseline echocardiography showed normal ventricular function (left ventricular ejection fraction 67%), and absolute neutrophil counts were within normal limits (5.9–7.8 × 10^9^/L). Broad‐spectrum antibiotic and antifungal therapy, directed at the pulmonary lesions given the possibility of infection, was reinstituted at the haematology‐oncology centre prior to initiation of myelosuppressive chemotherapy, though these lesions did not fully resolve. Ongoing IVIG replacement and standard supportive blood product transfusion were continued throughout treatment.

Following the second cycle of R‐CHOP, her clinical course was complicated by severe bilateral necrotising pulmonary infection. There was no evidence of pulmonary fibrosis or other acute pulmonary complications directly attributable to chemotherapy. Despite supportive management, the patient died approximately 2 months after diagnosis. The clinical timeline is summarised in Table 1.

**Table 1:** Clinical timeline.

Timepoint	Event	Category
Pre‐admission		
~2 weeks prior	Symptom onset	Clinical event
~12 days prior	Oral amoxicillin‐clavulanate antibiotics (private clinic)	Treatment
~9 days prior	IV ceftriaxone × 7 days (ambulatory)	Treatment
Inpatient—Farwaniya Hospital		
Day 0	Emergency admission; empiric antibiotics	Clinical event
Day 2–5	Persistent periorbital oedema and erythema; transient improvement on Day 5.	Clinical event
Day 8	MRI orbit: phlegmon 2.4 × 2.1 × 2.5 cm; MDT convened	Investigation
Day 9	Surgical intervention: I&D + FESS	Treatment
Day 11	Fever recurrence; abscess recollection 1.9 × 1.4 cm, antifungal initiated.	Clinical event
Day 14	Repeat incisional biopsy; antimicrobial and antifungal escalation.	Treatment
Day 17	Primary histopathology: B‐cell non‐Hodgkin lymphoma (CD20 + , CD79a + , BCL − 2+; Ki − 67*%*–80%); referred for reference review	Diagnosis
Postdiagnosis—Paediatric Oncology/Haematology Centre		
~Wk 1	Transfer; staging CT: pulmonary masses, mediastinal nodes, abdominal soft tissue disease	Investigation
~Wk 2 (Day 31)	Reference review: DLBCL, inborn error of immunity–associated (CD20 + , CD79a + , PAX − 5+, BCL − 2+, MUM − 1+; CD10−, BCL − 6−)	Diagnosis
~Wk 2–3	Bone marrow: no morphological infiltration; light chain–restricted B‐cell population on flow cytometry; CSF negative; Lugano stage IV	Investigation
~Wk 3–4	R‐CHOP initiated (cyclophosphamide and vincristine at ~50% dose)	Treatment
**~**Wk 8	Death; severe bilateral necrotising pulmonary infection	Fatal outcome

*Note:* Day numbers are counted from admission (Day 0); weeks are counted from primary histopathological diagnosis (Day 17).

Abbreviations: CSF, cerebrospinal fluid; CT, computed tomography; DLBCL, diffuse large B‐cell lymphoma; FESS, functional endoscopic sinus surgery; I&D, incision and drainage; IV, intravenous; MDT, multidisciplinary team; MRI, magnetic resonance imaging; R‐CHOP, rituximab, cyclophosphamide, doxorubicin, vincristine, prednisolone.

## 3. Discussion

The co‐occurrence of A‐T and orbital DLBCL in a paediatric patient represents an exceptionally rare clinical intersection. Although A‐T confers a substantially elevated risk of NHL, with large retrospective cohorts reporting primary cancer rates of 16.5%–24.7% and NHL predominating (55%–57%), typically as high‐grade mature B‐cell lineage with a cumulative incidence of 29% by age 35—paediatric orbital lymphoma itself is exceedingly rare, estimated at 0.12 per 1,000,000 children under 18 years. Within ocular adnexal lymphomas, DLBCL accounts for only 9.1% of cases yet carries the worst prognosis, with 60% disease‐specific mortality reported in the Surveillance, Epidemiology and End Results (SEER) cohort. Despite this well‐documented malignancy risk, heightened clinical suspicion remains the only available safeguard in the absence of a validated surveillance strategy [7, 10, 11].

Orbital lymphoma may present with periorbital swelling, proptosis, ptosis or visual changes—manifestations that are nonspecific and readily misattributed to infectious or inflammatory processes. This diagnostic challenge is further compounded in immunocompromised children, in whom immune dysregulation can produce a clinical picture indistinguishable from orbital cellulitis [8, 12]. In our patient, despite surgical drainage and escalating antimicrobial coverage, the swelling progressively enlarged and fever persisted, a clinical course that ultimately prompted biopsy and established the diagnosis. The underlying immune dysregulation of A‐T renders such presentations particularly convincing, a pattern mirrored in another reported case of orbital lymphoma in an A‐T patient, who similarly presented with dacryocystitis before a delayed diagnosis of Burkitt lymphoma [8]. These cases collectively underscore that malignancy must remain part of the differential in any immunocompromised child with a refractory orbital infection, regardless of how convincingly infectious the initial picture appears.

Establishing the diagnosis in this case was complicated by several converging factors. Clinically and radiologically, the lesion closely resembled complicated infectious dacryocystitis, and the initial MRI was interpreted as sinusitis with medial extraconal phlegmon, an attribution that deferred oncological consideration and delayed biopsy [4–6]. A fundamental constraint was the patient′s underlying radiosensitivity: defective ATM‐mediated DNA double‐strand break repair renders exposure to ionising radiation a meaningful risk of further mutagenesis, thus orbital CT was withheld [13, 14]. Serial imaging was consequently restricted to MRI and ultrasound, modalities specifically recommended in A‐T on account of their freedom from ionising radiation exposure, a principle increasingly formalised in the monitoring of children with DNA instability syndromes [15–17]. Whilst clinically appropriate, this restriction narrowed the diagnostic field and prolonged the interval to tissue diagnosis. Cross‐sectional imaging for staging introduces a further dilemma: while ionising radiation carries heightened genotoxicity risk, its use for accurate systemic staging is indispensable for treatment stratification; in our patient, diagnostic urgency rendered its use unavoidable. Even once obtained, however, the resulting staging CT could not reliably distinguish lymphomatous pulmonary involvement from widespread necrotising infection given her immunocompromised state, illustrating that imaging alone is frequently insufficient to separate malignant from infectious disease in this population. This case underscores that tissue biopsy with immunohistochemistry is the definitive diagnostic modality when orbital lesions fail to respond to standard management in immunocompromised children, and that proceeding to biopsy should not be unnecessarily delayed [4, 18].

Management of DLBCL in A‐T presents distinct challenges, owing to the heightened susceptibility to chemotherapy toxicity and radiosensitivity. The largest multinational cohort to date demonstrated that treatment‐related mortality (TRM) rather than disease progression is the primary cause of treatment failure, with a 4‐year cumulative incidence of 25.9%; patients with absent ATM kinase activity were disproportionately affected (TRM 37.6% vs. 4.0%, *p* = 0.017) [19]. Paediatric mature B‐cell lymphoma is conventionally treated with intensive LMB (lymphomes malins B)‐based regimens, with or without rituximab; in this patient, however, the treating team selected R‐CHOP as a less intensive backbone, reflecting both local resource allocation and the marked chemotherapy sensitivity described in A‐T, particularly to cyclophosphamide and vincristine [19, 20]. The latter also provided the rationale for dose reduction of cyclophosphamide and vincristine to approximately 50% of standard, consistent with consensus recommendations attributing excessive toxicity to defective ATM‐mediated non‐homologous end‐joining [19, 20]. Rituximab was incorporated into the dose‐modified backbone to preserve antitumour efficacy while limiting cytotoxic exposure, an approach supported by consensus recommendations, paediatric data on rituximab combined with LMB‐based chemotherapy and multiple case reports in A‐T‐associated mature B‐cell lymphomas [20–25]. Access to granulocyte colony‐stimulating factor, and to the novel targeted agents reported in A‐T‐associated lymphoid malignancies [26], was likewise constrained by local resource availability; R‐CHOP therefore represented the best available therapeutic option under these circumstances. Radiation was omitted entirely given the radiosensitivity of A‐T cells and associated risks of leukoencephalopathy and secondary malignancy [20, 27]. Despite these modifications, the fatal pulmonary infection in our patient, occurring after the second treatment cycle, illustrates that infectious complications remain a persistent and potentially lethal threat in the setting of baseline immunodeficiency and pre‐existing respiratory disease, both independent predictors of TRM [19, 20]. Treatment‐induced myelosuppression may also have contributed; however, as contemporaneous haematological data were unavailable, this could not be confirmed. This case underscores the absence of a standardised, A‐T‐specific treatment protocol, with published experience confined to case reports and small retrospective series applying empirical modifications to standard paediatric lymphoma regimens; nonetheless, upfront dose reduction of selected cytotoxic agents, omission of radiotherapy and aggressive infectious prophylaxis currently form the cornerstones of management [20, 28].

## 4. Limitations

This report has several limitations. Cytogenetic testing for MYC, BCL2 and BCL6 rearrangement was requested as part of the confirmatory pathology workup, but results were not available before the patient′s death; double‐hit or triple‐hit status therefore could not be excluded, and, as MYC immunohistochemistry was not performed, double‐expressor status also could not be assessed; both carry distinct prognostic and, potentially, therapeutic implications in DLBCL that could not be accounted for in this report. EBV status could not be determined: immunohistochemistry for EBV was not performed, as the relevant antibody was not available in Kuwait, and EBER in situ hybridisation was not performed; this is of particular relevance given the recognised role of EBV in inborn error of immunity–associated lymphomas. Treatment following diagnosis was delivered at a separate referral centre, and detailed haematological data from this period, including neutrophil counts at the onset of the fatal pulmonary infection, were not available to the authors; whether the infection occurred during chemotherapy‐induced neutropenia therefore could not be determined. Finally, as a single case report, the generalisability of these findings is inherently limited, and management decisions reflect the judgement of the treating multidisciplinary team rather than a standardised, evidence‐based protocol.

## 5. Conclusion

This case highlights that even well‐established infectious diagnoses warrant reassessment when the clinical course is refractory, particularly in the context of an underlying immunodeficiency syndrome carrying substantial malignancy risk. Although orbital DLBCL in a child with A‐T is exceptionally rare, no validated surveillance strategy currently exists, and malignancy must remain part of the differential regardless of how convincingly infectious the initial picture appears. Ionising radiation should be avoided where possible in A‐T given the underlying radiosensitivity but may be justified when essential for staging or treatment planning; MRI and ultrasound remain the preferred first‐line alternatives, though they too are nonspecific. When a mass fails to resolve with standard treatment, biopsy with immunohistochemistry is the definitive diagnostic step and should not be deferred. Once lymphoma is confirmed, management is further complicated by heightened chemotherapy toxicity, the omission of radiotherapy, a disproportionate risk of TRM compared with immunocompetent patients and the persistent threat of infectious complications driven by underlying immunodeficiency.

## Author Contributions

Fajer Alabdulrazzaq: writing—original draft, writing—review and editing, investigation and project administration. Arwa Alkanderi: writing—original draft and investigation. Bashayer AlHazeem: writing—original draft, writing—review and editing and investigation. Abdullah Alenezi: writing—original draft and investigation.

## Funding

No funding was received for this manuscript.

## Disclosure

All authors have read and approved the final version of the manuscript. Fajer Alabdulrazzaq had full access to all of the data in this study and takes complete responsibility for the integrity of the data and the accuracy of the data analysis. Fajer Alabdulrazzaq, as lead author, affirms that this manuscript is an honest, accurate and transparent account of the study being reported; that no important aspects of the study have been omitted; and that any discrepancies from the study as planned have been explained.

## Ethics Statement

This case report was conducted in accordance with the Declaration of Helsinki (1964) and its subsequent amendments and with applicable local and institutional guidelines. No experimental intervention was performed; the report constitutes a retrospective observational review of routine clinical care. The patient is fully de‐identified; no names, initials, or exact dates appear in the manuscript.

## Consent

Written informed consent for publication of this case report, including all clinical details and clinical photographs, was obtained from the patient′s mother and legal guardian. A copy of the signed consent form has been provided to the journal in confidence.

## Conflicts of Interest

The authors declare no conflicts of interest.

## Data Availability Statement

The data that support the findings of this study are available on request from the corresponding author. The data are not publicly available due to privacy or ethical restrictions.

## Supporting information


**Supporting Information** Additional supporting information can be found online in the Supporting Information section. File S1: CARE checklist of information to include when writing a case report.
